# Acceptability and implementation of a comprehensive digital diabetes self-management platform (MyWay Diabetes): a qualitative protocol

**DOI:** 10.1136/bmjopen-2025-105869

**Published:** 2025-06-25

**Authors:** Rhiannon E Hawkes, Dennis Ashpole, Mats Stage Baxter, Alex Bickerton, Hibbah Osei-Kwasi, Martin K Rutter, Daniel Taylor, Deborah J Wake, Tony Willis, Joanna Brooks

**Affiliations:** 1 Division of Psychology and Mental Health, The University of Manchester, Manchester, England, UK; 2 Patient and Public Involvement Contributor, Manchester, UK; 3 Usher Institute, The University of Edinburgh, Edinburgh, UK; 4 MyWay Digital Health Ltd, Dundee, UK; 5 Somerset NHS Foundation Trust, Somerset, UK; 6 School of Sport, Exercise and Health Sciences, Loughborough University, Loughborough, Leicestershire, UK; 7 Division of Diabetes, Endocrinology and Gastroenterology, The University of Manchester, Manchester, UK; 8 Manchester Diabetes Centre, Manchester University NHS Foundation Trust, Manchester, UK; 9 Vocal, Manchester University NHS Foundation Trust, Manchester, UK; 10 NHS North West London Integrated Care System, London, UK

**Keywords:** Self-Management, DIABETES & ENDOCRINOLOGY, Digital Technology, QUALITATIVE RESEARCH

## Abstract

**Abstract:**

**Introduction:**

Diabetes is one of the most common long-term health conditions worldwide, placing a huge economic burden on health services. Diabetes self-management education and support programmes can support people with diabetes to manage their condition; however, uptake of face-to-face services remains low. Digital self-management tools are becoming increasingly available. MyWay Diabetes is a digital platform that offers a comprehensive self-management and education programme accessible through a mobile app and website and allows patients to access their personal healthcare records. Following successful implementation in Scotland, MyWay Diabetes is now being rolled out in three geographical areas in England. We plan to undertake three qualitative studies, as part of a larger mixed-methods research programme, to assess whether MyWay Diabetes is acceptable across diverse patient groups and healthcare professionals and gather views of patients who do not currently use the digital service.

**Methods and analysis:**

We will conduct three online focus group studies. (1) One focus group with healthcare professionals (n=6–10) to understand their perceptions of implementing MyWay Diabetes in their local regions. (2) Up to four focus groups with existing users of MyWay Diabetes (n=24–40) across the three geographical areas in England to explore their acceptability of the platform. (3) Up to three focus groups with people living with diabetes who do not currently use MyWay Diabetes (n=18–30). Data will be collected using online videoconferencing and analysed thematically using template analysis.

**Ethics and dissemination:**

Ethical approval was granted by South Central – Berkshire Research Ethics Committee (ref: 25/SC/0125) and The University of Manchester Proportionate Research Ethics Committee (ref: 2025-23064-42006). Study results will be disseminated through peer-reviewed journals, conference presentations, MyWay Digital Health platforms and national bodies. The evidence from this broader mixed-methods evaluation will inform decisions for platform improvement and regional and national commissioning across the National Health Service in England.

**STRENGTHS AND LIMITATIONS OF THIS STUDY**

- This will be the first indepth qualitative assessment of user views of the MyWay Diabetes digital platform, across three geographical areas in England.
- This will be the first qualitative exploration of why people do not engage with MyWay Diabetes, and findings will be applicable to other digital self-management tools.
- We are working closely with our patient and public involvement and engagement group to shape the study design, participant recruitment, interpretation of findings and dissemination of findings to ensure this programme of work benefits from the perspective of people living with diabetes.
- Recruitment of participants will be restricted to those who speak and understand English due to the practicalities of taking part in an online focus group, but we will work closely with our patient and public involvement and engagement group and local organisations to recruit people from a wide variety of ethnic and socially diverse backgrounds, including people who speak English as a second language and those with an understanding of the challenges of language barriers (eg, through family members).

## Introduction

 Diabetes is one of the greatest health issues of the 21st century, with more than 1 in 10 adults living with diabetes globally.^1^ It is estimated that the global economic burden of diabetes will be more than US$2.1 trillion by 2030.^2^ In the UK, diabetes accounts for approximately 10% of National Health Service (NHS) spending, 80% of which is spent treating largely preventable complications.^3 4^ Approximately 90% of people living with diabetes in the UK have type 2 diabetes.^5^

Diabetes self-management education and support (DSMES) programmes can help people living with diabetes to manage their condition through providing guidance on diet, physical activity, glucose control and medication.^6 7^ DSMES programmes have been found to improve clinical and psychosocial outcomes and be cost-effective compared with usual care.^8 9^ The National Institute of Health and Care Excellence therefore recommends DSMES programmes be offered to all people newly diagnosed with diabetes, with annual reviews thereafter.^10^ However, recorded attendance at face-to-face sessions remains low globally,^7^ with only 7% of newly diagnosed patients with type 2 diabetes in the UK recorded as attending a session within their first year of diagnosis.^11^

Digital interventions have the potential to address some of the limitations of face-to-face programmes. For example, they may be more suitable for working adults, people with caring responsibilities, disabled people or people living in remote locations.^12^,^14^ Digital interventions for diabetes self-management can be effective, acceptable and promote patient autonomy.^6 15^ Such interventions are increasingly widely available; however, most of these are ‘stand-alone’ websites or apps with variable design and impacts, without integration with an individual’s clinical data. Integration with traditional care delivery has been suggested as a significant influencer of patients’ use and effectiveness of digitally-enabled diabetes self-management tools.^15^

MyWay Diabetes (MWD), developed by MyWay Digital Health, is an online platform that has been available in Scotland (as ‘My Diabetes My Way’) to people with diabetes and their carers since 2008. Since 2018, MWD has been available as a mobile application (app).^16^ Unlike other digital DSMES programmes, MWD provides a lifelong data tool to support self-management and offers users access to data available in their personal electronic health records used by their primary care clinicians. Access to these data aims to empower people with diabetes to better understand and manage their condition and thereby improve their health and reduce healthcare burden. More specifically, MWD comprises: (a) >250 multimedia education resources; (b) structured QISMET accredited eLearning; (c) an electronic personal health record through data-linkage with general practice and other healthcare IT systems; (d) personalised data-driven patient advice; (e) goal-setting functionality; (f) peer discussion forums; (g) integration with remote glucose/device monitoring systems and devices such as FitBit; and (h) a tool to help people better understand their risks and how to improve them.

Analysis of 2576 MWD users in Scotland showed HbA1c improvements within 1 year maintained for over ten years compared with matched non-users with diabetes.^17^ Extrapolation of Scottish data suggest that MWD could result in approximately £337 million NHS cost savings over 10 years if adopted by all people with type 2 diabetes across England (£84 million with 25% uptake).^17^ Since its development in Scotland, MWD is now also available in Somerset (MyWay Diabetes), Greater Manchester (Diabetes MyWay) and North West London (KnowDiabetes) in England.

### Acceptability of MyWay Diabetes

Medical Research Council guidance recognises the importance of evaluating the acceptability of healthcare interventions alongside assessment of programme effectiveness.^18^ For example, if key stakeholders (patients, carers and healthcare professionals) encounter barriers to using the programme, patients will not receive the potential benefits on offer, which will have knock-on effects on patient outcomes. Assessment of acceptability to stakeholders is therefore an important part of the MWD evaluation that could provide important insights into usability and uptake of the platform. This is particularly pertinent, given that recorded uptake of ‘My Diabetes My Way’ in Scotland is lower in people from ethnic minorities and those who live in more deprived areas.^19 20^

The Theoretical Framework of Acceptability (TFA)^21^ is a conceptual model outlining key components that influence how individuals perceive and accept a healthcare intervention. The framework has been developed to provide a coherent definition of acceptability within the context of health services research and identifies seven component constructs of acceptability: affective attitude, burden, perceived effectiveness, ethicality, intervention coherence, opportunity costs and self-efficacy.^21^ This framework has previously been used to assess the acceptability of digital and mobile interventions,^21 22^ including digital interventions for type 2 diabetes.^23^ The TFA will be used to inform the qualitative data collection and analysis in this programme of work.

In addition to exploring the acceptability of MWD for current users of the platform, it is also important to understand why people do not use MWD, which would also provide an understanding of the potential barriers to the uptake of digital DSMES programmes more widely. For example, a recent questionnaire with users of ‘My Diabetes My Way’ in Scotland found that some respondents mentioned a lack of digital skills to navigate the platform was a barrier to using the service.^16^ Further, diabetes care and subsequent outcomes are poorer among ethnic minority and socioeconomically disadvantaged groups, whom diabetes disproportionally affects.^24^ It is therefore important to understand why people might not engage with digital technologies and how digital self-management interventions, including MWD, can be designed to be more accessible and acceptable to more diverse populations. This has been called for by research evaluations of other nationally implemented diabetes programmes in England.^25 26^

This qualitative programme of work is nested within a broader mixed-methods research evaluation aiming to test the effectiveness of MWD. This qualitative research sets out to assess whether MWD is acceptable across diverse patient groups and healthcare professionals in Greater Manchester, Somerset and North West London, UK. This programme of work seeks to provide data to identify areas for platform improvement and assess whether MWD is suitable for regional and national commissioning across England. To achieve this aim, our objectives for this qualitative evaluation are to:

1. Assess usability and acceptability of MWD, in all participants and in diverse subgroups defined by age, ethnicity, socioeconomic status and geographical region.
2. Assess how MWD can be developed to improve equity.
3. Use the evidence generated to inform decisions for regional and national commissioning across the NHS in England.

## Methods and analysis

Data collection will start in June 2025 and analyses have a planned completion date of June 2026 for this qualitative work.

### Design

The research will involve the collection and analysis of data using a qualitative approach, which will address the study objectives across diverse populations where MyWay Diabetes or its derivatives have been deployed in England (Greater Manchester, Somerset and North West London). This will allow us to explore views and experiences of participants in an indepth way. To achieve this, we will conduct three studies:

1. Study 1: one online focus group and/or individual interviews with healthcare professionals to understand their perceptions of MWD, including any perceived barriers to participation and implementation.
2. Study 2: up to four online focus groups with existing MWD users to understand their perceptions of usability and acceptability of the platform, barriers to engagement and suggestions for how the service can be improved.
3. Study 3: up to three online focus groups with people across each of the three geographical regions who do not currently use MWD, to understand how to increase acceptability and usability of the platform for those who do not engage with digital self-management interventions.

### Participants

Healthcare professionals (HCPs) will meet the following inclusion criteria:

- Clinical leads, general practitioners with a special interest in diabetes, practice nurses, specialist diabetes nurses, healthcare assistants, hospital outpatient clinic staff, consultants, dieticians or other HCPs involved in diabetes care.
- HCPs who have been involved in the implementation of and/or the referral to MWD and are based in Greater Manchester, Somerset or North West London, UK.

Existing MWD users, representative of and reflecting diversity, will meet the following inclusion criteria:

- Aged 18 years or more.
- Diagnosed with diabetes.
- Are able to speak and communicate in the English language.
- Existing users of MyWay Diabetes, based in Greater Manchester, Somerset or North West London, UK.
- Existing users who have completed the consent form and screening/baseline characteristics form and fit the list of study inclusion criteria.

Patients who do not use MWD will meet the following inclusion criteria:

- Aged 18 years or more.
- Diagnosed with diabetes.
- Are able to speak and communicate in the English language.
- Live in Greater Manchester, Somerset or North West London, UK.
- Do not currently use MWD to self-manage their diabetes (either former users of MWD or patients who have never used MWD).

### Recruitment and sample size

#### Study 1: focus group with HCPs

We will conduct one online focus group with HCPs (n=6–10) representing services involved in diabetes care delivery from across a range of healthcare settings. See online supplemental material 1 for a flow diagram of recruitment of HCPs. We will consult within our wider project team to identify relevant HCPs through snowball sampling. Our collaborators in each geographical area are Clinical Leads for diabetes, who will contact relevant HCPs who meet our inclusion criteria via their professional networks. The Clinical Leads will also be eligible to take part themselves. Invitations to participate will come via contact from the Clinical Lead in their geographical area; the only exception is that the research team will also send each Clinical Lead an invitation via email as they are also eligible to participate.

With this invite, participants will receive an information sheet, and those who wish to take part can click on a link to an online consent form, hosted on the Qualtrics survey platform. Following completion of consent, participants will be asked to complete their availability for a selection of times and dates for a focus group and answer some demographic questions (job role, gender, geographical location) on Qualtrics. A focus group will then be run at the time that is most suitable to the majority of participants.

We will assess people’s characteristics (eg, job role, geographical area) in order to diversify the range of HCPs invited to participate in the focus group. This may mean that some people who have expressed an interest in participating are then unable to take part if they are: (a) unavailable at the time that the focus group has been scheduled and (b) if their characteristics are the same as other participants already confirmed to attend when we have other eligible participants who will contribute different characteristics to the group composition. Anyone who is not selected due to availability or the group already being full will be contacted by a member of the research team via email. If participants are not available to attend a scheduled focus group and we have recruited less than 10 participants to the group, they will be offered the option to take part in a one-to-one interview with the researcher, either over the telephone or via videoconferencing, at a time convenient for them. If participants initially provide consent but do not proceed to attend a focus group or interview, their data will be deleted from the Qualtrics database and researcher records.

#### Study 2: focus groups with existing MWD users

We will conduct up to four online focus group discussions with existing MWD users (n=24–40) to understand their perceptions of usability and acceptability of the platform, barriers to engagement and suggestions for how the service can be improved. See online supplemental material 2 for a flow diagram of recruitment of existing MWD users. Participants will be contacted by the digital service provider, MyWay Digital Health, who are the direct care team. The service provider will send an email invitation about the study via their mailing list (newsletters that are regionally based and sent to users). These are existing communications where users have already given their permission to be contacted by the provider. The email invitation will include a link to an information sheet, and interested participants will complete an online screening form hosted on the Qualtrics survey platform if they would like to be considered to take part in a focus group.

Interested participants will be asked to provide the following information: age, gender, ethnicity, length of diabetes diagnosis, postcode and contact details; these will only be available to the research team. Postcodes will only be used to identify deprivation indices using the UK Government’s postcode lookup for English Indices of Deprivation.^27^ The information gained from the screening questionnaire will be used to confirm eligibility for the study and to purposively select a sample with maximum variation and range of characteristics outlined above. After participants have submitted this information, they will be informed that a member of the research team will be in touch with them if they are shortlisted to take part in a focus group.

Those who are selected to proceed with participation in the focus groups will be sent a shortlisting email and sent a second link to an online consent form hosted on Qualtrics for completion, followed by some demographic questions (age, gender, ethnicity, duration of diabetes and postcode). Participants will also be able to select from a range of dates and times to attend an online focus group.

We are using a maximum variation sampling technique. This technique aims to maximise the variation of perspectives that we might be able to capture in our work by including in each of our groups people with a range of different backgrounds and characteristics. Participants will be selected initially based on diversity of demographic characteristics and then based on their availability to attend one of the groups, as described for Study 1, above. If a particular group is over-subscribed, we will assess people’s characteristics in order to diversify the range of people invited to participate in that group. Those selected for participation will be contacted by the researcher via email or telephone with details of the arranged focus group. Those not selected for focus group participation will be informed via email, which will include a debrief sheet. Their data will be deleted as soon as possible from the Qualtrics screening questionnaire database and researcher records.

#### Study 3: focus groups with patients who do not use MWD

See online supplemental material 3 for a flow diagram of recruitment of patients who do not use MWD. We will conduct up to three focus groups (n=18–30) with people living with diabetes who do not currently use MWD, to understand ‘prospective acceptability’,^21^ barriers to engagement and to gather insights into what facilitators need to be in place for diverse patient populations to take up and engage with MWD. We expect these insights will also be applicable to the non-take-up and non-use of other digital diabetes self-management tools.

We are working with study collaborators and our patient and public involvement and engagement (PPIE) group who have contacts with local community organisations and the Voluntary, Community, Faith and Social Enterprise (VCFSE) sector. Participants will hear about the research through a study advert, which will be distributed by trusted advocates within communities including distributing the adverts via WhatsApp groups through snowball sampling, and placed in community settings (eg, churches, mosques, community centres, faith-based organisations).

The study advert will include the contact details of the research team for interested people to get in touch for more information about the study and will include a link/QR code to the information sheet. The researcher will explain the study to each interested person, and with their permission, will take note of the person’s name and contact details and ask what days/times would be most convenient for a focus group. During this call, the researcher will also confirm the following eligibility questions: that the person is living with diabetes, they live in either Greater Manchester, Somerset or North West London and that they are not current users of MWD. Participants will be sent the information sheet again via email or post following the phone call.

We will use a maximum variation sampling technique as described for Studies 1 and 2, above. Those selected for a focus group will be contacted by the researcher and full verbal consent will be taken via telephone prior to the focus group. During the consent process, participants will also be asked some demographic questions (age, gender, ethnicity and postcode). Those not selected for focus group participation will be informed via telephone or email and will be sent a debrief sheet. Their data will be deleted as soon as possible from researcher records.

### Procedures

#### Study 1: focus group with HCPs

One focus group discussion will take place, lasting approximately 60 min. It will be held online (eg, via Zoom or Microsoft Teams) with 6–10 participants. Following a short presentation about MyWay Diabetes (including demographic characteristics of existing users), group discussion will explore (a) perceptions of MyWay Diabetes; (b) relevant target patient groups (eg, ethnicity, socioeconomic/education status, age); and (c) any perceived barriers to participation and/or implementation.

We have developed a broad topic guide for the focus group, informed by the TFA^21^ to explore aspects of acceptability of MWD for HCPs, and Normalisation Process Theory (NPT)^28 29^ to further explore the implementation of MWD. The NPT framework is well-established for identifying barriers and facilitators to implementing interventions, including digital/technology-enabled type 2 diabetes interventions.^30^

#### Study 2: focus groups with existing MWD users

The focus group discussions will be held online (eg, via Zoom or Microsoft Teams), lasting approximately 60 min. A flexible topic guide to investigate acceptability and any proposed modifications to MWD will be broadly structured around the constructs of acceptability delineated in the TFA^21^ and developed in collaboration with our PPIE group. Group discussions will explore how MWD has helped with their diabetes self-management and potential adaptations to improve the platform for diverse patient groups. After the focus group has taken place, participants will be emailed with a debrief sheet, including further details of organisations with support for their diabetes self-management. As a ‘thank you’, participants will be offered a Love-to-Shop voucher for taking part, in line with National Institute for Health and Care Research guidelines for payment to public contributors.^31^

#### Study 3: focus groups with patients who do not use MWD

On the day of the scheduled focus group, the researchers will check that all participants have previously provided consent over the telephone with the research team. Anyone who has not yet provided consent will be taken individually to a ‘break-out room’ and full verbal consent will be taken by an experienced member of the research team. Focus groups are planned to be held online (eg, via Zoom/Microsoft Teams), lasting approximately 60 min. From speaking with our collaborators, people in their networks are more open to online meetings since COVID-19. The researcher will ensure that participants feel confident about how to use the online videoconferencing platform and will offer a ‘practice’ online meeting with any individual who requests this prior to the focus group to ensure they feel more confident in taking part. If participants have issues with accessing the internet for an online focus group, they will be offered the option to take part in a telephone interview with the researcher at a time to suit them. We will keep the option open to conduct the focus group in-person in if this is the preference of trusted advocates/community leaders on behalf of their communities.

A flexible topic guide to investigate prospective acceptability of MWD and other digital technologies will be broadly structured around the constructs of the TFA^21^ and developed in collaboration with our PPIE group. Group discussions will explore perceptions of digital technologies, barriers to uptake of digital technologies and potential adaptations to improve accessibility and equity across diverse communities. After the focus group has taken place, participants will be emailed with a debrief sheet, including further details of diabetes support organisations and further signposting to free resources to help improve digital literacy skills (eg, ‘Learn My Way’ platform^32 33^). As a ‘thank you’, participants will be offered a Love-to-Shop voucher for taking part, in line with existing guidelines.^31^

### Data analysis

All focus groups will be audiorecorded (with the participants’ consent), transcribed and identifying information will be removed from the transcripts. The TFA theoretical framework^21^ will be applied to the analysis across the three studies to allow the comparison of constructs of acceptability across three stakeholder groups (HCPs, existing MWD users and those who do not use MWD). The NPT theoretical framework^28 29^ will also be applied the analysis of the HCP data to identify mechanisms that play an important role in the implementation of MWD in routine care.

Qualitative data from each of the three studies will be analysed thematically using template analysis, an approach that permits the use of a priori themes to structure the analysis around the constructs of TFA and NPT.^34^ We will use established quality-checking procedures, including an appropriate audit trail, critical scrutiny (including by PPIE contributors) and constant comparison of coding between analysts. Initial data coding across the three datasets will be undertaken using an inductive, data-driven approach to ensure that non-theory delineated factors are not overlooked and nuance and content not lost. Codes will be considered in relation to relevant theoretical domains and organised into meaningful clusters (including hierarchical and lateral relationships to facilitate understanding of how themes interrelate). A full thematic structure for each of the three datasets will be developed iteratively, and findings synthesised in later stages providing an overview of stakeholders’ views on MWD. We will use NVivo software to manage the data.

### Patient and public involvement and engagement

The original MWD platform was created and has been developed with extensive PPIE input, with those who have had involvement at every stage of the design, prototyping, development, implementation and review phases of the platform. MyWay Digital Health receives regular feedback from patients via email, secure messaging and web-based surveys (eg,^16^) to ensure that the platform is patient-centred, and holds regular steering group meetings which include representation by patients.

Throughout this programme of research, we will partner with Vocal,^35^ a not-for-profit organisation specialising in PPIE in health research. Vocal will: (a) support the inclusion of PPIE throughout key aspects of the research; (b) provide training to PPIE contributors; and (c) lead PPIE meetings that will contribute to decision-making within the project. One of our co-applicants is a person living with diabetes and a co-author of this protocol paper; he has worked closely with our representative from Vocal and members of the study team to shape the research during the early stages of design and will meet twice a month with the research team to ensure the overall management of the project benefits from a lived experience perspective.

With Vocal’s support, we have recruited seven additional public contributors from the three geographical areas where MWD is implemented (Greater Manchester, Somerset and North West London). Contributors were purposively recruited to ensure diverse patient views are prioritised. The PPIE group will meet online quarterly and will advise on all aspects of the research from the design (eg, focus group schedules, recruitment), interpretation of findings and dissemination of findings to wider audiences. They will be paid for their time in line with existing guidance.^31^ Their involvement and contribution throughout the research lifecycle will ensure that lived experience is accurately considered in the research design and conduct to optimise the relevance of the research to the wider community of people with diabetes.

## Ethics and dissemination

###  Ethical approval

Studies 1 and 2 have been reviewed and approved by South Central – Berkshire Research Ethics Committee (ref: 25/SC/0125). Study 3 has been reviewed and approved by The University of Manchester Proportionate Research Ethics Committee (ref: 2025-23064-42006). The research will be conducted in full conformance with all relevant legal requirements and the principles of the Declaration of Helsinki, Good Clinical Practice and the UK Policy Framework for Health and Social Care Research 2017. Identifying information will be removed from the focus group/interview transcripts and participants will be assigned pseudonyms.

### Consent

The online consent process on the Qualtrics survey platform for studies 1 and 2 will require participants to respond ‘yes/no’ to each statement before proceeding. Participants have the option to download their responses and completed consent form once their response is submitted. Participants will also have the option to complete verbal consent over the telephone with the researcher if: (a) they are having problems accessing the online form or (b) they feel more comfortable speaking with the researcher before providing full consent. For study 3, full verbal consent will be taken by an experienced member of the research team. The consent form will be read to the participant and they will be asked to confirm verbally if they agree to each statement. This will be audio recorded with the participant’s approval and recorded separately from the main focus group.

### Dissemination

Study results will be shared via peer-reviewed conference presentations and open-access peer-reviewed journal publications. Findings will be made publicly available to participants, PPIE contributors and to the general public via blogs, the MyWay Digital Health Ltd platforms/social media and national bodies, with appropriate recognition of study participants’ contributions.

At the end of the project, we will deposit a fully anonymised dataset (anonymised transcripts) in an open data repository where it will be permanently stored. We will use Figshare at the University of Manchester Library. Researchers at other institutions and others can access the anonymised data directly from the repository and use it for further research, to check our analysis and results or for teaching purposes. Participants will be informed in the information sheet that the transcripts will be fully anonymised and will not identify them or be combined with other information in a way that could identify them.

### Anticipated outcomes

This qualitative study will generate robust evidence describing the usability, acceptability and implementation of MWD as a comprehensive diabetes self-management platform. The evidence from this broader mixed-methods evaluation will be generated to inform decisions for regional and national commissioning across the NHS in England.

### Summary and conclusion

This will be the first in-depth qualitative assessment of user views of the MyWay Diabetes digital platform and the first exploration of why people do not engage with it. Evidence from this evaluation will inform ongoing development of the platform, decisions for platform improvement and regional and national commissioning across the NHS in England.

## Supplementary material

10.1136/bmjopen-2025-105869 online supplemental file 1
