# Supplementary material for: Acceptability and implementation of a comprehensive digital diabetes self-management platform (MyWay Diabetes): a qualitative protocol
Source: BMJ Open. 2025 Jun 25;15(6):e105869. doi: 10.1136/bmjopen-2025-105869 (PMC12198844; doi:10.1136/bmjopen-2025-105869)

Supplementary Material 1: Summary of healthcare professional recruitment (study 1)

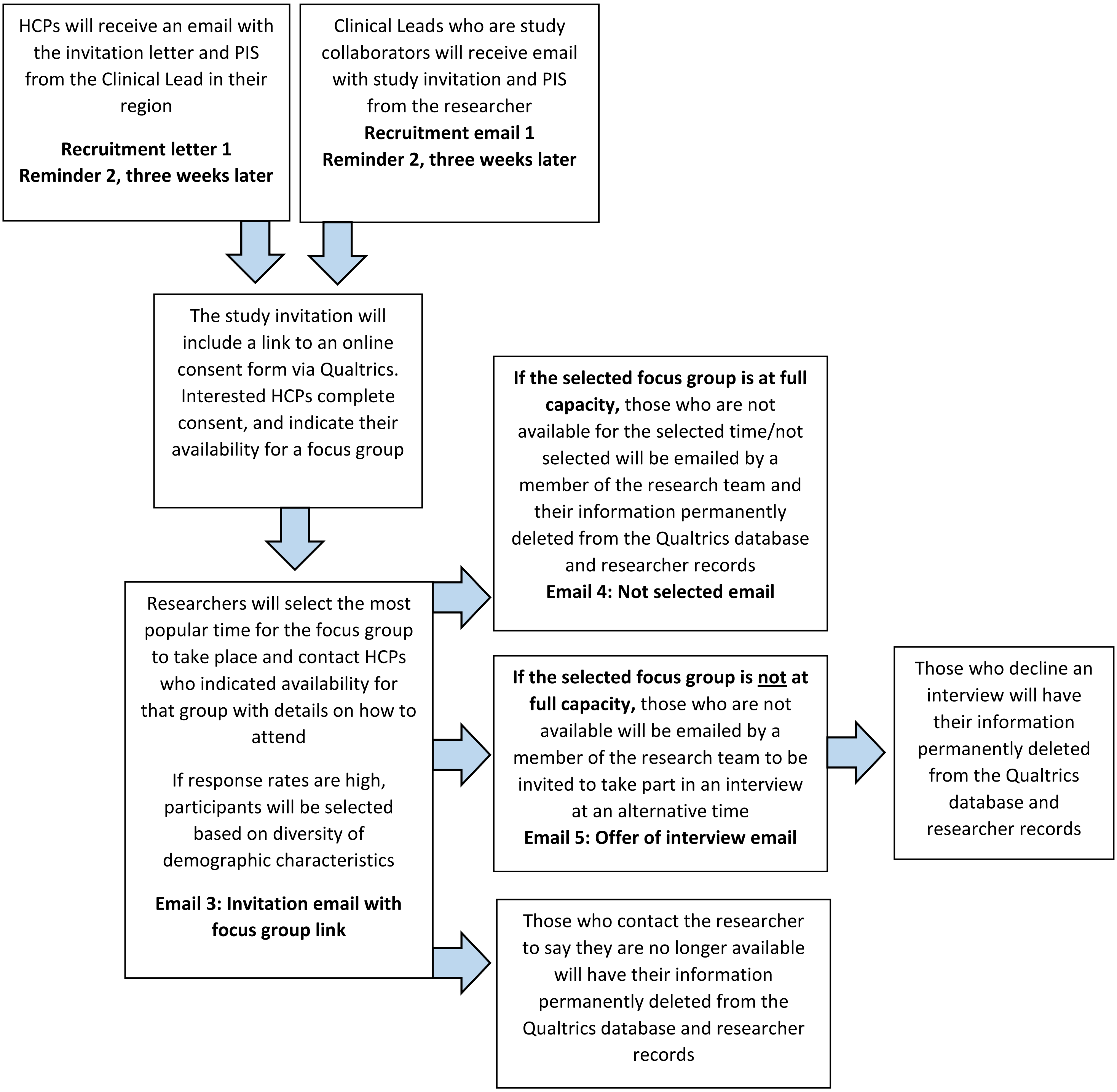

Supplementary Material 2: Summary of existing MyWay Diabetes user recruitment (study 2)

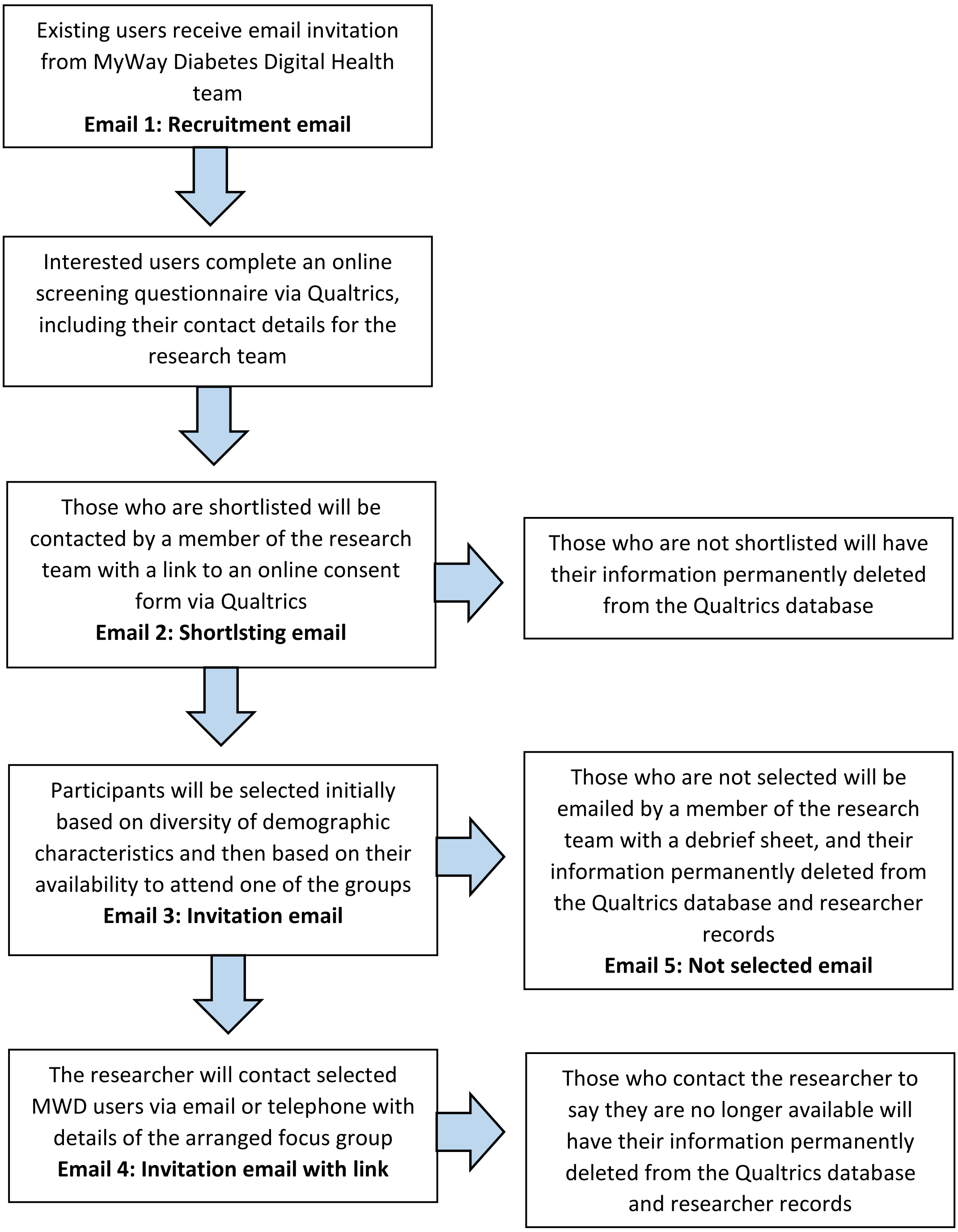

Supplementary Material 3: Summary of recruitment of patients who do not use MyWay Diabetes (study 3)

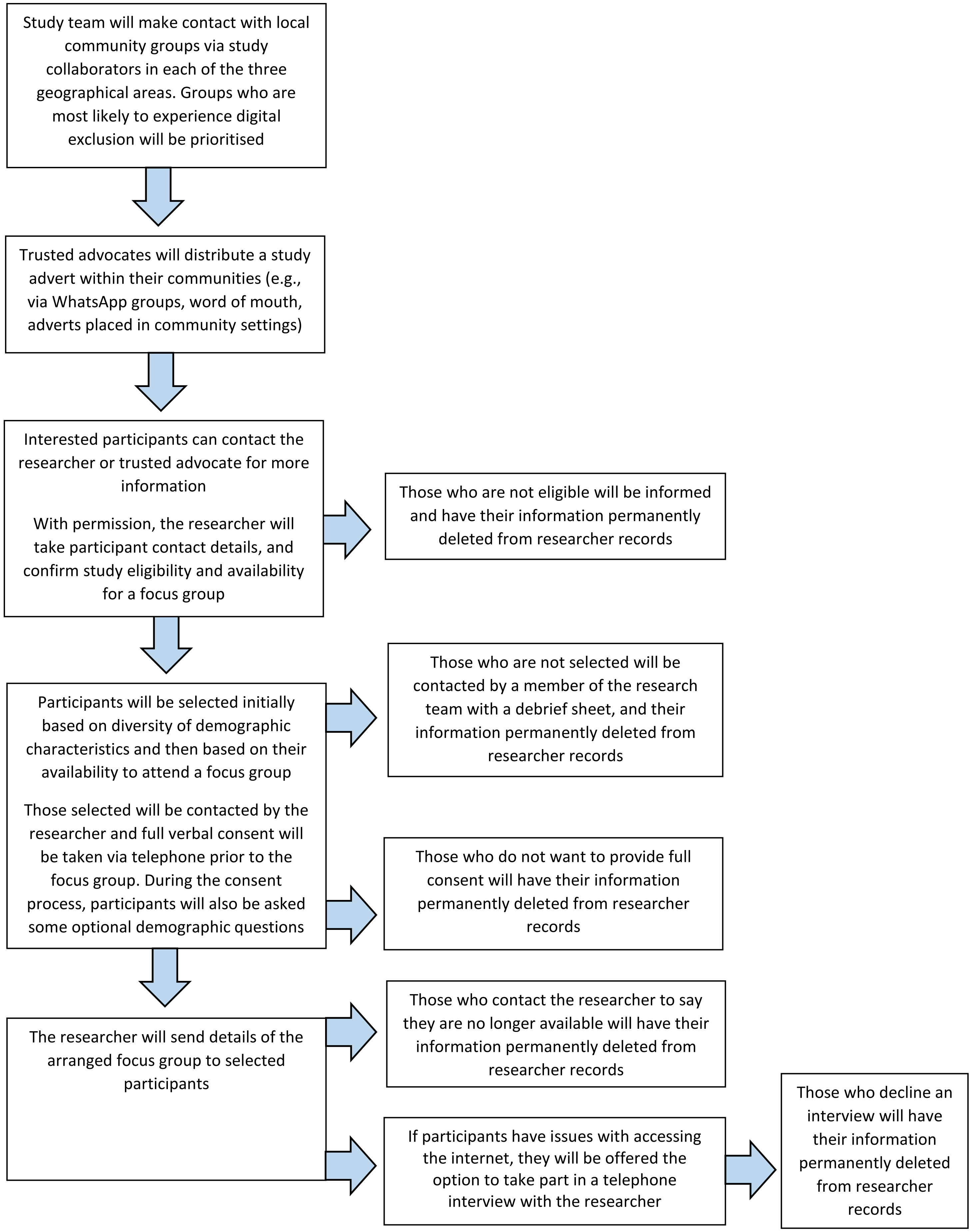

Supplement: online supplemental file 1 [file bmjopen-15-6-s001.pdf]
