# Supplementary material for: Acceptability and implementation of a comprehensive digital diabetes self-management platform (MyWay Diabetes): a qualitative protocol
Source: BMJ Open. 2025 Jun 25;15(6):e105869. doi: 10.1136/bmjopen-2025-105869 (PMC12198844; doi:10.1136/bmjopen-2025-105869)
Supplement: Supplementary file 2 [file bmjopen-2025-105869.reviewer_comments.pdf]

PEER REVIEW HISTORY

BMJ Open publishes all reviews undertaken for accepted manuscripts. Reviewers are asked to complete a checklist review form and are provided with free text boxes to elaborate on their assessment. These free text comments are reproduced below.

ARTICLE DETAILS

Title (Provisional)

Acceptability and implementation of a comprehensive digital diabetes self-management platform (MyWay Diabetes): A qualitative protocol

Authors

Hawkes, Rhiannon Eleri; Ashpole, Dennis; Baxter, Mats Stage; Bickerton, Alex; Osei-Kwasi, Hibbah; Rutter, Martin K; Taylor, Daniel; Wake, Deborah J; Willis, Tony; Brooks, Joanna

---

This article was not externally reviewed at BMJ Open. Protocols that have been independently assessed prior to submission to BMJ Open are usually fast-tracked to publication on the grounds that further substantial changes will not be possible. This independent assessment will usually be external, independent review for both a substantial grant award from a non-commercial or government funder and ethics approval.
